# Correction of Long-Lasting Negative Effects of Neonatal Isolation in White Rats Using Semax

**Published:** 2012

**Authors:** M.A. Volodina, E.A. Sebentsova, N.Yu. Glazova, D.M. Manchenko, L.S. Inozemtseva, O.V. Dolotov, L.A. Andreeva, N.G. Levitskaya, A.A. Kamensky, N.F. Myasoedov

**Affiliations:** Institute of Molecular Genetics, Russian Academy of Sciences; Biological Faculty, Lomonosov Moscow State University

**Keywords:** chronic stress, neonatal isolation, Semax, body weight, corticosterone, rat

## Abstract

Adverse experience during the early postnatal period induces negative alterations in physiological and neurobiological functions, resulting in long-term disorder in animal behavior. The aim of the present work was to study the long-lasting effects of chronic neonatal stress in white rats and to estimate the possibility of their correction using Semax, an analogue of ACTH fragment (4–10). Early neonatal isolation was used as a model of early-life stress. Rat pups were separated from their mothers and littermates for 5 h daily during postnatal days 1–14. The pups of the control group were left undisturbed with the dams. Half of the rats subjected to neonatal isolation received an intranasal injection of Semax at a dose of 50 µg/kg daily, from postnatal day 15 until day 28. The other animals received intranasal vehicle injections daily at the same time points. It was shown that neonatal isolation leads to a delay in physical development, metabolic disturbances, and a decrease in the corticosterone stress response in white rats. These changes were observed during the first two months of life. Semax administration weakened the influence of neonatal isolation on the animals, body weight , reduced metabolic dysfunction, and led to an increase in stress-induced corticosterone release to the control values. So the chronic intranasal administration of Semax after termination of the neonatal isolation procedure diminishes the negative effects of neonatal stress.

## INTRODUCTION

It is well known that the neonatal period of life is very important in the neurophysiological mechanisms development and the subsequent formation of mental functions. Aversive experience during the early postnatal period of human life (such as parental loss, abuse or parental neglect) results in an increase of psychopathology development probability in adulthood [1]. Children who had severe diseases in the neonatal period are exposed to painful and stressful influences, resulting in acute changes and permanent alterations in the structure and functions of the central nervous system [2]. Although the correlation between neonatal stress and behavioral disorders in adults has been demonstrated by a number of researchers, more study of the question are required. Animal experiments using various aversive actions enable to determine the relationship between the delayed changes in behavior, duration, and the type of actions, as well as to facilitate the search for methods for correcting the effects of neonatal stress. It has been demonstrated in numerous clinical studies that a disturbance of the socio-emotional mother-infant relationship during the first year of life is a significant stressor, which subsequently increases the risk of a number of mental disorders development [1, 3]. Long-term maternal isolation in the early postnatal period (neonatal maternal deprivation) also influences the behavior and physical development of various animal species.

There are a lot of studies devoted to the investigation of the long-lasting effects of neonatal maternal deprivation (MD). It has been demonstrated that the delayed effects of chronic MD depend on the duration of the daily deprivation of pups. Short-term chronic deprivation (15 min per day during the first 1–2 weeks of life) has a positive effect on the subsequent development of the animals. The rats that underwent such experience showed reduced anxiety and increased exploratory activity, as well as learning ability improvement [4–6]. Long-term separation of the rat pups from their mother (for 3–6 h per day during the first few weeks of life) also causes long-lasting delayed changes in animal behavior and is considered to be the neonatal stress model. Two models of maternal deprivation are used in the experiments. In the first case, the pups of the same litter stay together during MD. In the second case, the pups are subjected to neonatal isolation (NI): the pups are placed into individual boxes, where they are separated both from their dams and littermates. An increase in the anxiety level and reduced exploratory activity were observed in most experiments in the animals subjected to MD [7–9]. Nevertheless, MD occasionally resulted in an increase in the animals exploratory activity [10]. The effect of long-term MD on the animals, learning ability is also controversial. Different researchers have detected both disturbance [11, 12] and improvement of the spatial learning ability in maternally deprived animals [13]. In some studies, no effect of MD on the spatial learning ability of rats was observed [14]. The influence of MD and NI on the functioning of the hypothalamic-pituitary-adrenal axis was demonstrated. However, the results were appreciably controversial, similar to those in the case of the animal behavior alteration. Thus, whereas some authors reported an increase in stress-induced corticosterone release in mice that were subjected to maternal deprivation [15, 16], others reported a decrease in this index in animals that were subjected to NI [17–19] or MD [20]. In a number of studies, no changes in the hormonal stress-response in animals that had experienced neonatal stress were revealed [7]. The inconsistency of the results could be due to the differences in the experimental protocols and in the age of the tested animals [18]. Thus development of an adequate neonatal stress model in animals and further study of the delayed effects of chronic long-term maternal deprivation is quite essential.

The heptapeptide Semax (MEHFPGP) is an ACTH fragment (4–10) analogue that has prolonged neurotropic activity [21]. This peptide possesses neuroprotective and neurotrophic effects [22, 23]; it also has antihypoxic and antihaemorrhagic action [21, 24]. Semax is used in medicine as a nootropic and neuroprotective agent [25]. It was demonstrated earlier that chronic neonatal administration of Semax results in enhancement of the exploratory behavior and a decrease in anxiety in rats. Moreover, the animals that received Semax in the neonatal period showed better learning ability in subsequent years. These alterations had a delayed long-lasting character [26]. Semax neonatal administration effects were opposite to the effects of neonatal stress: that let us to assume that Semax administration can correct the negative effects of neonatal stress. It was ascertained in our previous studies that the daily neonatal isolation of rat pups for 5 h during 1–2 weeks of postnatal development causes long-lasting alteration in the animal’s behavior. An increase in anxiety and reduction in exploratory activity at the age of 1–2 months was observed in rats subjected to NI during the first weeks of postnatal development. Chronic intranasal administration of Semax during 15–28 days of life resulted in a considerable normalization of the emotional state of the animals exposed to NI [27].

The aim of the present work is to study the effects of neonatal isolation on physical development in rats and the hormonal stress-response, as well as to find out whether Semax administration during 15-28 postnatal days can correct the NI effects.

## EXPERImental

Noninbreded white rat pups of both sexes were used. The animals were housed in a vivarium under the standard conditions with free access to food and water and were maintained on a 12 h light–dark cycle. The heptapeptide Semax (MEHFPGP) was synthesized in the Institute of Molecular Genetics, Russian Academy of Sciences.

The day of birth of the pups was considered as day 0 of life. Each litter was divided into three groups: the control group, the NI group (the animals were subjected to neonatal isolation), and the NI–Semax group (the animals were subjected to NI and subsequently received Semax). The pups from the control group were left undisturbed in their nest for the first two weeks of life. The pups from the NI and NI–Semax groups were daily placed into individual boxes for 5 h (days 1–14 of life). During the isolation, the pups were left in silent conditions at a temperature of 25 ± 2°С, and they were illuminated with moderate light. The rats from the NI–Semax group intranasally received a 0.05 mg/kg dose of an aqueous Semax solution during the period from day 15 to day 28 of life. The pups from the control and NI groups received an equivalent volume of the solvent during the same time period. During the experiment, the age of eye-opening and body weight were recorded for each animal (daily during the period from day 15 to day 28 of life; then, once a week). Blood glucose levels were measured at days 15, 30, and 48 of life. Blood samples were obtained from the tip of the tail in order to determine the glucose level; the glucose content was measured using a glucometer (Accu-Chek Performa Nano).

The level of food motivation in animals was assessed on day 42 of life. Prior to the experiment, the animals were deprived of food for 20 h. The blood glucose level was measured in hungry animals; after 30 min had elapsed, the rat was placed into an empty cage. After 5-min adaptation to the new conditions, a weighed portion of food was placed into the cage. Then, the following parameters were recorded during 10 min: the latency to food intake, feeding duration, and the amount of food consumed. Next, the rat was placed in a cage with unlimited access to food. The glucose level was measured again after 30 min.

**Fig. 1:**
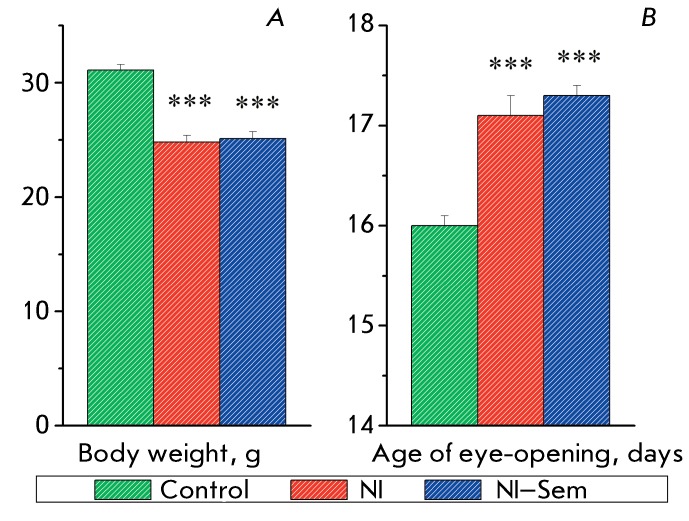
Neonatal isolation effects on the body weight of rats at the age of 15 days ( *A* ) and on the time of eye-opening ( *B* ). The number of animals in groups: 81/74/77. *** (p < 0.001) – significant difference from the control.

**Fig. 2:**
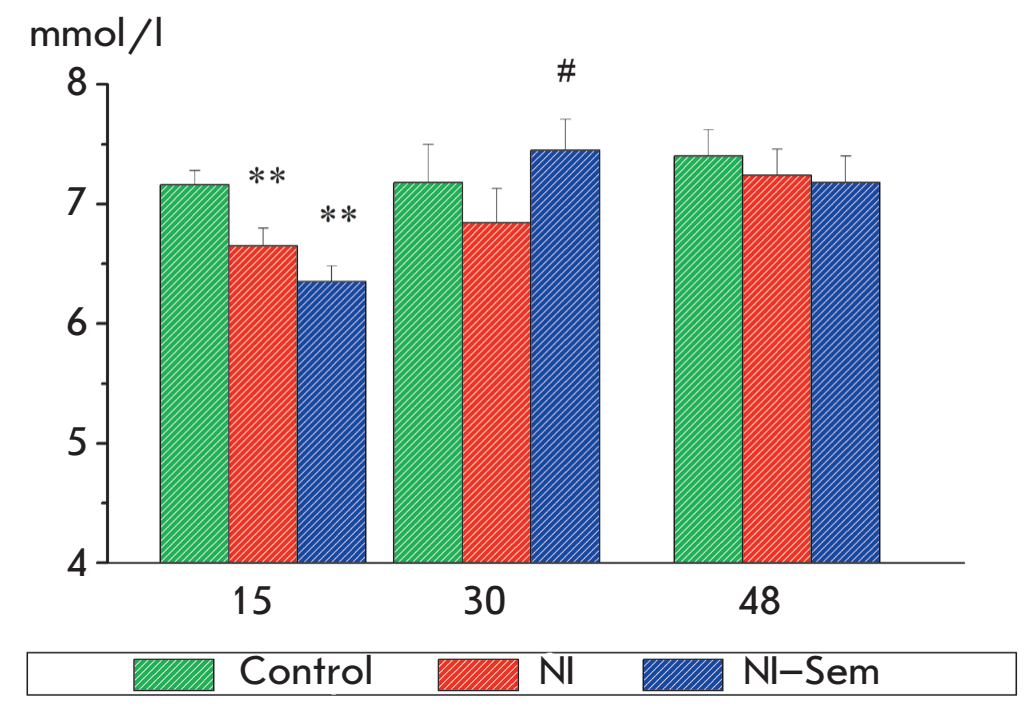
Blood glucose level in rats. The x-axis – the age of animals (days); the y-axis – glucose concentration (mmol/l). The number of animals in groups: rats at the age of 15 days – 38/35/38; rats at the age of 30 and 48 days – 12/11/11. **(p < 0.01) – significant difference from the control, #(p < 0.05) – significant difference from the NI group.

The alteration in corticosterone in rat blood as a response to an acute stressor was assessed on day 65 of life. In the beginning of the experiment, a rat was placed into an immobilizing device; a blood sample (200 µl) was obtained from the tail after cutting off the tip. The animal was subjected to forced swim stress at 24°С for 10 min. Ten min after the termination of the stressor exposure, the rat was repeatedly placed into the immobilizing device. The second blood sample was obtained; the animal was then placed back into its home cage. Sixty min after the termination of the forced swim stress, the rats were decapitated to obtain a blood sample. The sample was kept at 37°С for 20 min, and at 4°С for 60 min. The samples were then centrifuged (10 min, 5,000 rpm), and the serum was collected. The corticosterone level was subsequently assessed in the serum samples using the Corticosterone EIA Kit designed to determine corticosterone in biological fluids (Catalog № ADI-900-097, Enzo).

## RESULTS

Animals of both sexes were used in the experiments. The factor Sex had a considerable effect only on the body weight alteration of the rats. Other parameters did not differ significantly in either males or females. It was shown via use of the two-way ANOVA method (factor 1 –Group; factor 2 – Sex) for analyzing the alteration in the body weight of the rats during the first two months of life that the Group ( *F*_2,48_  = 3.49, *p * < 0.04) and Sex factors ( *F*_1,48_  = 34.91, *p * < 0.000001) had a significant effect. However, no significant interaction between these factors was observed ( *F*_2,48_  = 0.33, *p * = 0.72). No statistically significant differences in the influence of NI and Semax on animals of different sexes were revealed by comparison with the results obtained for the male and female groups. This fact allows us to present the results obtained for the entire group of rats.

It was demonstrated that daily isolation during the first two weeks of life results in body weight decrease in 15-day-old rat pups ( *F*_2,229_  = 39.60, * p * < 0.0001; *Fig. 1A* ) and delay in eye-opening *(F*_2,136_  = 25.83, *p*  < 0.0001; *Fig. 1B* ) versus animals of the control group. In addition, a significant decrease in the blood glucose level was detected 1 day after the last NI in rats that had experienced neonatal stress in comparison with those in the control group ( *F*_2,107_  = 9.53, *p * < 0.0001; *Fig. 2* ). On day 15 of life, the pups subjected to NI were randomly divided into the NI and NI–Semax groups. The animals from these two groups had identical body weight, glucose level at day 15 of life, and the age of eye-opening ( *Figs. 1, 2* ).

Half of the pups exposed to NI received a daily intranasal Semax injection during the period from postnatal day 15 to day 28 (the NI–Semax group). The remaining animals (the NI group) and the animals from the control group received distilled water. The lower body weight in rats from the NI and NI–Semax groups was observed during this period as compared to those in the control group ( *F*_2,84_  = 27.75, *p*  < 0.0001; *Fig. 3A* ). No significant differences between the NI and NI–Semax groups were detected ( *F*_1,53_  = 0.03, *p * > 0.85). The animals from the NI group had a lower body weight in comparison with that of the rats in the control group up to day 65 of life ( *F*_1,25_  = 4.63, *p*  < 0.04). Until day 48, the body weight of the rats in the NI–Semax group remained significantly lower than that in the control group. No significant differences were subsequently detected ( *F*_1,26_  = 2.87, * p*  > 0.10; *Fig* . *3B* ).

**Fig. 3:**
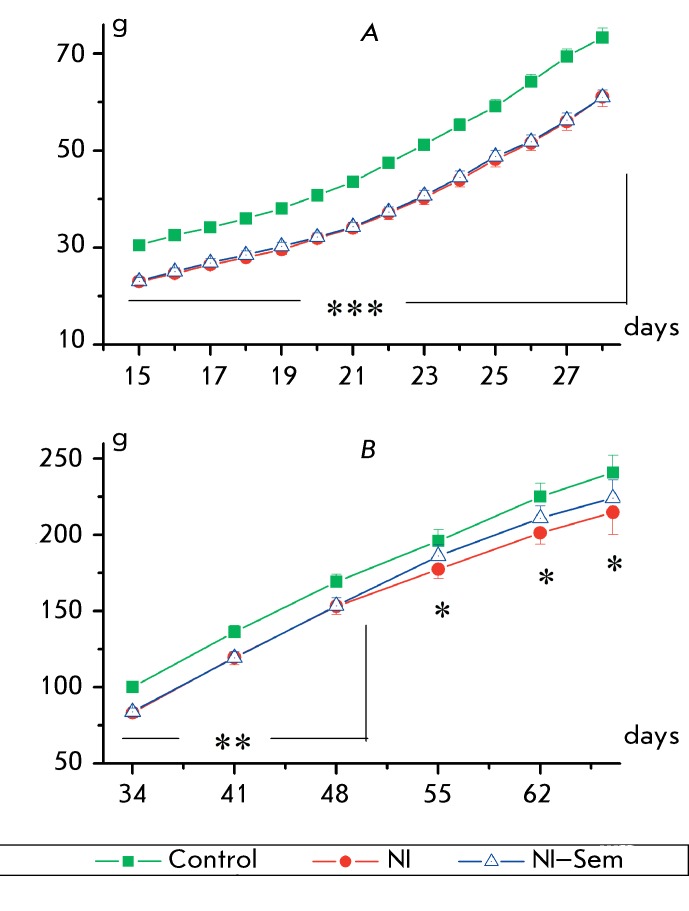
The growth rate of rats during the first (A) and the second (B) months of life. The x-axis – the age of animals (days), the y-axis – the body weight (g). The number of animals in groups: 32/29/28. *(p < 0.05), **(p < 0.01), and ***(p < 0.001) significant difference from the control.

**Fig. 4:**
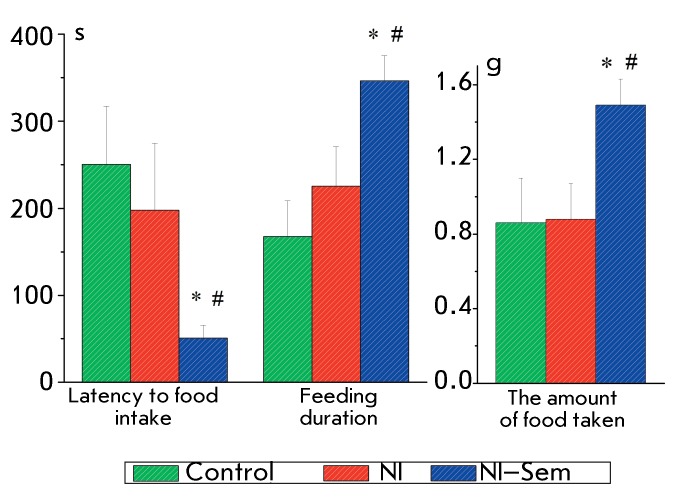
The results of food motivation test for the rats at the age of 42 days. The rats were food-deprived for 20 h before testing. The number of animals in groups: 12/11/11. *(p < 0.05) – significant difference from the control, #(p < 0.05) – significant difference from the NI group.

No significant differences in the blood glucose level in 30-day-old rats were revealed between the groups ( *F*_2,32_  = 1.09, *p*  > 0.25), although the blood glucose content in the pups from the NI group was lower as compared to the control values. Subsequent analysis demonstrated that the value of this parameter in the NI–Semax group was significantly higher than that in the NI group ( *p * < 0.02 using the χ ^2 ^ test). No differences in the glucose level were observed for the groups of 48-day-old rats ( *F*_2,31_  = 0.74, *p*  > 0.50) ( *Fig. 2* ).

The food motivation level of the animals was assessed on day 42. In the rats from the NI group, the parameters characterizing the food motivation level were identical to those in the control group. The group of animals receiving Semax injections demonstrated a reduction of the latency to food intake, an increase in feeding duration and the amount of food consumed during the experiment, as compared to these parameters in the control and NI groups ( *F*_2,31_  > 3.3, *p * < 0.05) ( *Fig. 4* ). The aforementioned changes attest to increased food motivation in the NI–Semax animals. Hence, the neonatal isolation experience did not affect the food motivation level of rats; Semax administration to the rat pups exposed to NI resulted in an increase in food motivation.

It was demonstrated that the blood glucose level in NI rats after 24-h food deprivation was significantly lower as compared with that in the control and NI–Semax groups ( *F*_2,31_  = 3.32, *p * < 0.05). The glucose level after food deprivation in NI–Semax animals was identical to that in the control group. The repeated measurements (after the food intake) showed no significant differences in this parameter between the groups ( *F*_2,31_  = 0.46, *p*  > 0.60) ( *Fig. 5* ). Thus, neonatal isolation resulted in the reduction of the blood glucose level under conditions of food deprivation. Semax administration eliminated the NI effect in case of this parameter.

**Fig. 5:**
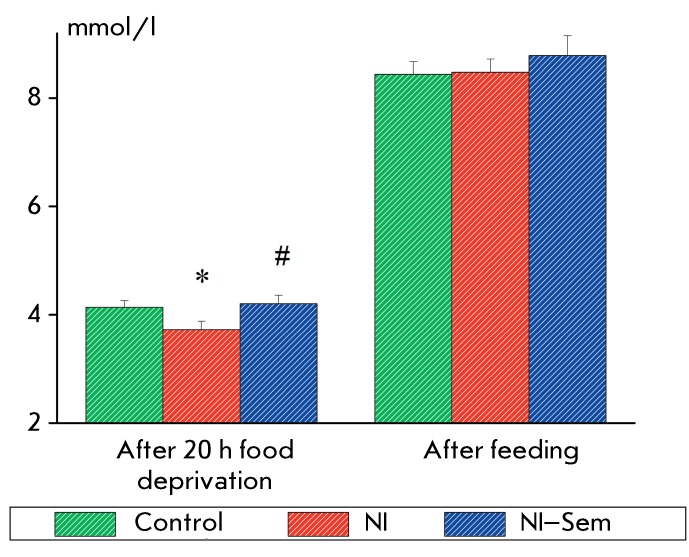
Blood glucose level in rats subjected to 20-hour food deprivation (before and after food consumption). The y-axis – glucose concentration (mmol/l). The number of animals in groups: 12/11/11. *(p < 0.05) – significant difference from the control, #(p < 0.05) – significant difference from the NI group.

**Fig. 6:**
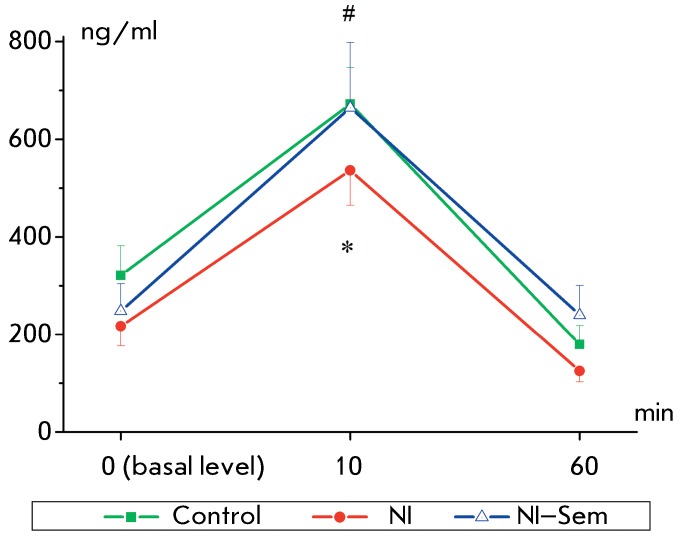
Stress-induced changes in corticosterone level: basal (0), 10 and 60 min after exposure to stress. The y-axis – serum corticosterone concentration (ng/ml). The number of animals in groups: 13/12/11. *(p < 0.05) – significant difference from the control, #(p < 0.05) – significant difference from the NI group.

The changes in the blood corticosterone level of the rats in response to a acute stressor were assessed at day 65 of life. The corticosterone basal level in NI rats was lower than that in the control group; however, this difference did not attain the level of statistical significance ( *p*  > 0.05). Ten minutes following stress termination, the blood corticosterone level in NI rats was significantly lower than that in the control and NI–Semax groups ( *p*  < 0.05 using the χ ^2^ test). No significant differences between the groups with respect to this parameter were obtained 1 h after stress termination ( *Fig. 6* ). Thus, neonatal isolation had resulted in a reduction in the stress-induced corticosterone release; whereas Semax administration had eliminated the isolation effect, bringing the corticosterone level to the control value.

## DISCUSSION

The rat pups were isolated from their mothers and littermates for 5 h daily during the period from day 1 to day 14 of life. It was established earlier that rodents exhibit a weak response of the hypothalamic-pituitary-adrenal system to moderate stress during the period of early neonatal development (the period known as period of stress hyporeactivity). Ensured by specific maternal behavior, the suppression of the stress response plays a significant role in the normal development of the nervous system. It was demonstrated that the isolation of the pups from their mothers weakens the blockage of the hypothalamic-pituitary-adrenal system [28, 29]. NI procedure in the present work included daily food deprivation for 5 h, cold stress, and the absence of contact with the mother; so a combination of physical and emotional stress of high intensity was used. Furthermore, as mentioned above, the separation of the rat pups from their mothers resulted in a weakening of the neonatal stress-hyporeactivity. Thus, the actions used were stressors of high intensity. The model used can be considered to be a neonatal stress model.

It was demonstrated that chronic NI of rat pups during the period from day 1 to day 14 of life resulted in delayed eye-opening, which attests to the delayed physical development. The observed effects of NI are similar to those of MD [30, 31]. Changes in body weight is another parameter characterizing the physical development of the animals. In the model used, NI resulted in delayed somatic growth of the rat pups. The differences between the body weight of the NI rats and the control rats remained during the entire experiment time; i.e., at least up to the age of 2 months. There is a lack of consistency in the published data relating to the effect of MD on the body weight of the animals. In most of the studies, no changes in this parameter were detected [32, 33]. However, it was mentioned in a number of studies that the pups that had undergone MD had a reduced body weight [19, 34, 35]. The difference in the effects can presumably be attributed to the different conditions under which the pups were kept during the deprivation. Thus, it was demonstrated that NI at a temperature of 30°С did not affect the body weight of the animals, whereas NI at 22°С resulted in a decrease in body weight and growth deceleration [36]. In our experiments, during the NI procedure the pups were kept at 24–26°С. Presumably, a decrease in body temperature caused by the isolation of the pups from their mother and littermates plays a significant role in the development of the NI effects on their physical development. In addition, it was demonstrated that maternal deprivation causes suppression of the cell response to three major trophic hormones (growth hormone, prolactin, and insulin). These changes may result in somatic growth deceleration [37].

Thus, the daily isolation during the first two weeks of life resulted in the deceleration of the physical development of rat pups, which remained up to day 65 of life. Intranasal Semax injections to the 3- to 4-week-old rat pups weakened the effect of NI on the body weight of the animals; thus, these parameters approached the control level at days 55–65. This compensatory action of peptides on rat body weight is presumably based on the increased food motivation of the stressed animals that received Semax. In our case, the increased food motivation can be considered to be the adaptive response of the organism to the reduction in body weight caused as a result of neonatal isolation.

A significant decrease in the blood glucose level was recorded in rats that had experienced neonatal isolation at the age of 15 days. The glucose level was measured 24 h after the last isolation procedure; i.e., by the time the blood sample was collected, the rat pups had been in contact with their mothers for 1 day. Therefore, the decrease in the blood glucose level that was observed cannot be explained by food deprivation. The glucose level was not significantly different from the control values in the NI animals with unlimited access to food at the age of 30 and 48 days. However, under conditions of food deprivation, decreased glucose content was observed in NI rats compared to that in the control group. The resulting data attest to the fact that neonatal isolation causes long-lasting disturbances in metabolic processes in the rat organism. Semax administration to the animals exposed to NI resulted in an increase in the blood glucose level under conditions of both unlimited access to food and food deprivation, attesting to the fact that the peptide has a normalizing effect. It is well-known that it is necessary to maintain a physiological blood glucose level for the normal development of the brain in mammals. During the period when the nervous system is under development, hypoglycaemia may cause disorders both in cognitive functions and in the emotional status. These disorders do not disappear after the glucose level is normalized; they can manifest themselves in adulthood [38]. It was previously shown that NI during the first 1–2 weeks of life causes long-lasting behavioral changes: 1- to 2-month-old rats that had experienced NI demonstrated increased levels of anxiety and reduced exploratory activity. Subsequent Semax administration normalized the emotional state of the NI animals [27]. The abatement of NI-induced metabolic disturbances may have been one of the mechanisms of the positive effect of Semax on the emotional status of the animals that had experienced neonatal stress.

Study of the neonatal stress effect on the corticosterone level in blood demonstrated that NI had no effect on the basal level of this hormone; however, it resulted in a decrease in corticosterone release as a response to acute stress exposure. No effect of MD on the basal level of corticosterone had been observed in most of the previous studies [33, 39]. There has been no consistency in the published data relating to the effect of MD on stress-induced corticosterone release. It should be noted that most studies have been devoted to the investigation of the effects of maternal deprivation rather than neonatal isolation; this could account for the inconsistency in the results. Rees *et al.* [18] compared the effects of MD and NI and demonstrated that whereas MD did not affect the basal and stress-induced corticosterone release, NI resulted in a decrease in the stress-induced corticosterone release. The reduced hormonal response to stress in animals that had experienced neonatal isolation may be caused by the exposure to repeated stress episodes, which may have led to the reiterated release of corticosterone. The repeated activation of the hypothalamic-pituitary-adrenal system during the early neonatal period may result either in depletion of this system or in an increase in the efficiency of negative feedback [17, 35]. Semax administration to the rats subjected to NI boosted the level of stress-induced corticosterone release to the control level. Therefore, the subsequent administration of the peptide normalized the hormonal response to stress exposure, which had been disturbed by neonatal isolation.

It was previously demonstrated that daily neonatal isolation of white rat pups for 5 h during the period from day 1 to day 14 of life results in long-lasting changes in animal behavior [27]. It has been shown in this study that the neonatal exposure to stress also results in delay of physical development in the animals, disturbance of metabolic processes, and weakening of the hormonal response to acute stress. These changes were observed during the first 1–2 months of life; i.e., they were of delayed and long-term character. The negative effects of the neonatal stress were reduced by the chronic intranasal administration of Semax after the termination of the procedure of neonatal isolation. The results obtained can be used to broaden the range of clinical applications of Semax; in particular, for the treatment of pathologies in children during the early postnatal period. 

## Abbreviations

ACTH adrenocorticotropic hormone; MD: maternal deprivation
NI: neonatal isolation
